# Genetic features of precursor B‐cell phenotype Burkitt leukemia with IGH‐ *MYC* rearrangement

**DOI:** 10.1002/cnr2.1545

**Published:** 2021-09-02

**Authors:** Masanori Yoshida, Daisuke Tomizawa, Satoshi Yoshimura, Tomoo Osumi, Kazuhiko Nakabayashi, Hiroko Ogata‐Kawata, Keisuke Ishiwata, Aiko Sato‐Otsubo, Yui Kimura, Shuichi Ito, Kimikazu Matsumoto, Takao Deguchi, Nobutaka Kiyokawa, Takako Yoshioka, Kenichiro Hata, Motohiro Kato

**Affiliations:** ^1^ Department of Pediatric Hematology and Oncology Research National Center for Child Health and Development Tokyo Japan; ^2^ Department of Pediatrics Yokohama City University Yokohama Japan; ^3^ Children's Cancer Center National Center for Child Health and Development Tokyo Japan; ^4^ Department of Maternal‐Fetal Biology National Center for Child Health and Development Tokyo Japan; ^5^ Department of Pediatrics, Graduate School of Medicine The University of Tokyo Tokyo Japan; ^6^ Department of Pathology National Center for Child Health and Development Tokyo Japan

**Keywords:** *FBXO11*, IGH‐*MYC*, *KRAS*, preBLL

## Abstract

**Background:**

An atypical form of Burkitt leukemia/lymphoma (BL), BL with a phenotype of precursor B‐cells (preBLL), is listed in the WHO Classification. Recent reports suggested that preBLL and classical BL could be distinguished by the differences in IG‐*MYC* translocation architecture and an additional mutated genes profile. The characteristics of classical BL are IG‐*MYC* by aberrant somatic hypermutation or class switch recombination, and BL‐specific gene mutations such as *MYC*, *ID3*, and *CCND3*. Meanwhile, preBLL is characterized by IG‐*MYC* due to aberrant VDJ recombination and mutations in *NRAS* and *KRAS*. However, it is not clear whether all preBLL cases can be differentiated. This report investigated the molecular characteristics of an infant preBLL case, with a more advanced stage of maturity than typical preBLL.

**Case:**

The patient showed BL‐like morphology with IGH‐*MYC* rearrangement. In the immunophenotyping, CD20 and surface immunoglobulin were negative, whereas other markers were consistent with BL. To evaluate the genetic contribution, we performed whole‐exome sequencing. The breakpoint analysis revealed the IG‐*MYC* occurred due to an aberrant VDJ recombination. Meanwhile, additional somatic mutations were detected in *FBXO11*, one of the mutant genes specific to BL. In the analysis of the specimen in complete remission, mutation in *KRAS*, frequently mutated in preBLL, was detected with low frequency, suggesting somatic mosaicism.

**Conclusion:**

The present case showed the characteristics of both typical preBLL and classical BL. Because preBLL includes atypical cases such as the present case, further studies are required to elucidate preBLL features.

## 1 INTRODUCTION

IGH and *MYC* translocation is one of the representative genetic abnormalities in Burkitt lymphoma/leukemia (BL). Generally, BL shows a mature B‐cell phenotype. However, in the WHO Classification, a more immature phenotype of BL (preBLL) is listed as a phenotype of precursor B‐cells, with expression of terminal deoxynucleotidyl transferase (TdT), and sometimes CD34, and absence of CD20 and surface immunoglobulin expression.^1^ Recently, Wagener et al. reported that preBLL and classical BL could be distinguished from each other by differences in the translocation architecture of IGH‐*MYC*.^2^, ^3^, ^4^ They reported that aberrant V (variable), D (diversity), and J (joining) gene segments recombination resulting in IG‐*MYC* characterized by N‐sequences and loss of some bases at the breakpoint are features of preBLL. They also revealed a difference in mutation profile between preBLL and BL. PreBLL was associated with *NRAS* and/or *KRAS* mutations whereas BL was associated with *MYC*, *ID3*, and *CCND3*. Subsequently, Yoon et al. showed that IGH‐*MYC* translocations due to the aberrant VDJ recombination were also detected in the TdT‐negative cases, which indicated that these cases were at a more advanced maturation stage than those of Wagener et al.^5^ Their cases were associated with mutations in *MYC* and/or *TP53*, which are often mutated genes in BL.

Therefore, preBLL and BL have been found to have different genetic features, including IGH‐*MYC* translocation architecture. Meanwhile, it is still unclear at which maturation stage preBLL and BL can be distinguished, due to the limited number of reported cases. We here present an infant preBLL case with IGH‐*MYC* translocations due to the aberrant VDJ recombination despite revealing BL‐specific genetic abnormalities, suggesting a more advanced maturation stage than previous reports.

## 2 RESULTS

### 2.1 Clinical course of the case

A 10‐month old boy was admitted with a 2‐week history of ill complexion and fever. Physical examination showed head masses and bilateral renal swelling. A laboratory examination revealed thrombocytopenia (5.5 × 10^4^ platelets/μl) and total leukocyte count of 11 400/μl with blast cells with basophilic cytoplasm containing vacuoles. Flow cytometric immunophenotyping of blast cells was positive for cyCD22 (98.6%), cCD79a (99.7%), CD19 (99.0%), CD10 (97.9%), CD24 (99.1%), cyμ (91.5%), CD22 (98.8%), HLA‐DR (99.5%), and CD38 (99.8%), and negative for CD20, μ, κ, λ, CD34, and TdT (Table S1). The expression of CD45 was bright. Karyotyping analysis showed 46, XY, dup(1)(q21q32)x2,t(8;14)(q24;q32) [3]/46, XY [17]. Fluorescence in situ hybridization analysis on blast cells identified the fusion signal of IGH‐*MYC*, leading to a diagnosis of Burkitt leukemia with B‐cell phenotype (detailed in Figure S1). Morphological findings, negativity for CD34 and TdT, and positivity for cyμ and CD45 were suggestive of mature B‐cell phenotype, whereas negative for CD20 and surface globulins were immature B‐cell features (preBLL). Cerebrospinal fluid examination showed an increase in the number of dysmorphic cells (40/μl) with a high N/C ratio, nucleus with irregular shapes, and distinct nucleoli, and was cytologically determined as class V. Computed tomography revealed bilateral renal swelling, hepatomegaly, and splenomegaly. As the present case had both B‐cell precursor and mature B‐cell features of ALL, we considered that both lymphoma‐oriented intensive block‐type chemotherapy and ALL‐oriented chemotherapy, including maintenance therapy, should be adopted. Although the present case was infant ALL, we did not adopt infant‐specific chemotherapy such as in MLL‐10,^6^ which did not include block‐type treatment. Therefore, the present case was treated according to AIEOP‐BFM ALL 2000 high‐risk chemotherapy.^7^ Despite suspending prednisolone for 2 days due to tumor lysis syndrome, the patient showed a good prednisolone response. The patient achieved a complete remission (CR) after induction therapy. Considering that the patient was an infant, intensification of intrathecal chemotherapy was adopted to avoid the late complications of cranial radiotherapy. As of 38 months from diagnosis, the patient is alive without disease relapse.

### 2.2 Identification of additional somatic mutations and IGH‐*MYC* translocation architecture

**FIGURE 1:**
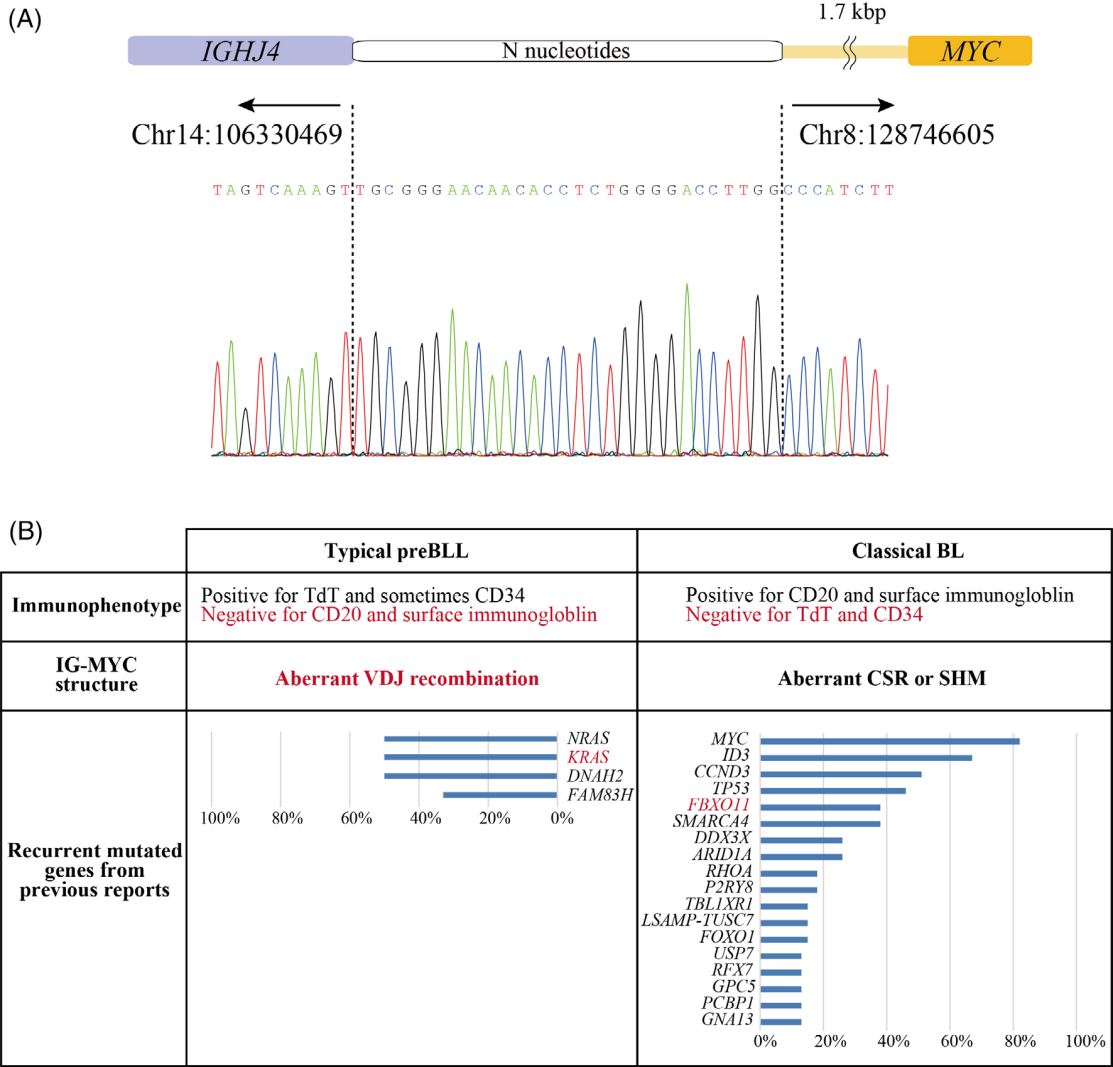
Genetic characterization of the case. (A) Translocation architecture of IGH‐*MYC* breakpoint junctions. The breakpoint mapped to the 5′ end of IGHJ4, with loss of three bases from the 5′ end, and 1.7 kbp upstream of *MYC*. Nucleotides resembling N‐sequences were added at the breakpoint. (B) The summary of the present case's characteristics along with a comparison with typical preBLL and BL characteristics with the reference to previous report of Wagener et al.^3^ The findings of typical preBLL and classical BL that are consistent with the present case are marked in bordeaux color. The recurrent mutated genes were listed with reference to previous genetic analysis of preBLL and classical BL.^3^, ^4^ BL, Burkitt lymphoma/leukemia; CSR, class switch recombination; preBLL, B‐cell phenotype Burkitt leukemia; SHM, somatic hypermutation

To reveal the present case's genetic characteristics, we further performed whole‐exome sequencing of tumor‐normal pairs (detailed in Data S1). We filtered out the variants that were present in normal sample, and identified two somatic mutations in *FBXO11* and one in *TNIK* (Table 1). Visual screening using the integrative genomics viewer (IGV) viewer (IGV 2.3.97) revealed that the two mutations in *FBXO11* were located on different alleles (Figure S2). Next, we focused on the breakpoint junction of the IGH‐*MYC* translocation using the IGV. The breakpoint mapped to the 5′ end of IGHJ4, with loss of some bases from the 5′ end, and 1.7 kbp upstream of *MYC* (Figure 1A). As the breakpoint was close to recombination signal sequences and nucleotides resembling N‐sequences were added at the breakpoint, the translocation most likely occurred through an aberrant VDJ recombination.

**TABLE 1:** Somatic mutations detected by whole‐exome sequencing

Gene	Region	AA change	SIFT	PP2_HDIV	CADD
*FBXO11*	Splicing	NM_001190274:exon14:c.1797+1G>A			28.5
*FBXO11*	Exonic	NM_001190274:exon14:c.A1713C:p.L571F	T^a^	P^b^	24.7
*TNIK*	Exonic	NM_001161566:exon24:c.G2978A:p.R993Q	D^c^	D^d^	35

Abbreviation: PP2, PolyPhen 2.

^a^ Tolerated.

^b^ Possibly damaging.

^c^ Deleterious.

^d^ Probably damaging.

### 2.3 Identification of germline variants of cancer‐predisposing genes

As the present case developed atypical leukemia in infancy, we further searched for germline pathogenic variants of cancer‐associated genes (detailed in Data S1). As a result, a pathogenic variant of *KRAS* (c.G34C, p.G12R), which has been registered as a somatic mutation in NCBI ClinVar and Catalog of Somatic Mutations in Cancer (COSMIC), was identified with a suspected mosaic mutation frequency (variant allele frequency = 19%) in a peripheral blood sample during CR. This *KRAS* variant was also identified in tumor samples. To obtain the precise frequency of mutation alleles, droplet digital PCR procedures were performed using QX‐200 and ddPCR™ Mutation Assay: *KRAS* p.G12R (Bio‐Rad, Hercules, CA). The mutation allele frequency was 9.5% in the CR sample and 46% in the tumor sample, respectively. The mosaicism could not be validated in any other organ due to the lack of available samples. The summary of the present case's characteristics is shown in Figure 1B, along with a comparison with typical preBLL and BL characteristics.

## 3 DISCUSSION

Wagener et al. and López et al. recently reported the features of IG‐*MYC* translocation architecture in preBLL and BL, respectively.^3^, ^4^ They showed that preBLL and BLL were well distinguished from each other by IG‐*MYC* translocation architecture and mutated genes. However, more recently, Yoon et al. reported TdT‐negative preBLL cases carrying IG‐*MYC* due to aberrant VDJ and additional mutations in BL‐specific genes.

In addition to being negative for TdT and CD34, the present case was positive for cyμ, and expression of CD45 was bright. These immunophenotypic profiles indicated a more advanced maturation stage of preBLL than in those of Yoon et al.^5^ The translocation architecture of IGH‐*MYC* in the present case showed the characteristics of preBLL, whereas additional mutations were identified in *FBXO11*. *FBXO11* is considered as a tumor suppressor gene in the pathogenesis of B‐cell lymphomas.^8^, ^9^ Mutation in *FBXO11* was identified in diffuse large B‐cell lymphoma and BL.^4^, ^10^ Additionally, a point mutation in *TNIK* was also identified. TNIK is one of the enzymes associated with Wnt signaling and is associated with its activation.^11^ As the *TNIK* mutation identified in the present case was not previously reported, the pathogenicity is unknown. It might have contributed to the oncogenesis. Meanwhile, mutations in other BL‐specified genes, such as *MYC* and *ID3*, were not detected in the present case. These atypical patterns of additional mutated genes might be responsible for the differences in the maturation stages of preBLL.

As another genetic abnormality, *KRAS* mutation, p.G12R, was detected in a CR sample with a suspected mosaic mutation frequency. p.G12R is one of the hot spot somatic mutations in *KRAS* registered in COSMIC. Germline and/or somatic mosaicisms of *KRAS* are shown to be associated with developmental disorders, such as Noonan syndrome.^12^ In the process of B‐cell development, KRAS contributes to early B‐cell development at the pre‐B‐cell stage and late B‐cell maturation.^13^ As *KRAS* p.G12R is a gain‐of‐function missense mutation, the oncogenic *KRAS* may have been responsible for the abnormalities in preBLL development and maturation in the present case. In support of that, the percentage of *KRAS* mutations was greater in tumor samples than in CR samples. As the *KRAS* mutation was mosaic, the patient would have not showed the symptoms characterizing Noonan syndrome. This finding indicates the importance of germline specimen analysis to evaluate the effect of genetic background on the development of childhood cancer.

Although our case fortunately achieved sustained remission, optimal therapeutic strategy of preBLL has not yet been established. In recent studies comparing treatment outcomes of preBLL, mature B‐cell NHL type chemotherapy is encouraging because of the favorable outcome.^14^, ^15^ Further genetic studies for this unique subset might provide us a clue to the best strategy against preBLL.

## CONFLICT OF INTEREST

The authors have stated explicitly that there are no conflicts of interest in connection with this article.

## AUTHOR CONTRIBUTIONS

All authors had full access to the data in the study and take responsibility for the integrity of the data and the accuracy of the data analysis. *Conceptualization, data curation, formal analysis, investigation, validation, visualization, writing—original draft*, M.Y.; *Conceptualization, resources, supervision, writing—review and editing, D.T.; Data curation, formal analysis, investigation, validation*, S.Y.; *Conceptualization, resources*, T.O.; *Data curation, investigation, methodology, software*, K.N.; *Data curation, investigation, methodology, software*, H.O.‐K.; *Data curation, investigation, methodology, software*, K.I.; *Investigation, methodology, visualization*, A.S.‐O.; *Resources*, Y.K.; *Supervision*, S.I.; *Resources, supervision*, K.M.; *Data curation, investigation, methodology*, T.D.; *Data curation, investigation, methodology, supervision*, N.K.; *Investigation, methodology, resources, visualization*, T.Y.; *Methodology, software, supervision*, K.H.; *Conceptualization, funding acquisition, methodology, project administration, resources, supervision, writing—review and editing*, M.K.

## ETHICAL STATEMENT

Genomic analysis was approved by the Institutional Review Board of the National Center for Child Health and Development (#1035), and the required informed consent was obtained from the guardians of the patient.

## Supporting information


**Figure S1.** Morphologic, immunocytologic, and cytogenetic features of leukemic blasts Peripheral blood smear reveals increased blast cells with basophilic cytoplasm containing vacuoles (A). In immunostaining, the blasts were positive for CD10 (B), CD19 (C), CD79a (D), and c‐Myc (G), and negative for CD20 (E) and TdT (F). Fusion FISH of IGH‐*MYC* was positive (H).
**Figure S2.** Genomic location of the two FBXO11 mutations Visual screening using the IGV viewer revealed that the two mutations in ***FBXO11*** were on different alleles (in trans).
**Table S1.** Flow cytometry findings at diagnosis.

## Data Availability

The data that support the findings of this study are available on request from the corresponding author. The data are not publicly available due to privacy or ethical restrictions.
