# Supplementary material for: Genetic features of precursor B‐cell phenotype Burkitt leukemia with IGH‐ MYC rearrangement
Source: Cancer Rep (Hoboken). 2021 Sep 2;5(7):e1545. doi: 10.1002/cnr2.1545 (PMC9327653; doi:10.1002/cnr2.1545)
Supplement: Supplementary file 1 — Figure S1. Morphologic, immunocytologic, and cytogenetic features of leukemic blasts Peripheral blood smear reveals increased blast cells with basophilic cytoplasm containing vacuoles (A). In immunostaining, the blasts were positive for CD10 (B), CD19 (C), CD79a (D), and c‐Myc (G), and negative for CD20 (E) and TdT (F). Fusion FISH of IGH‐MYC was positive (H). Figure S2. Genomic location of the two FBXO11 mutations Visual screening using the IGV viewer revealed that the two mutations in FBXO11 were on different alleles (in trans). Table S1. Flow cytometry findings at diagnosis. [file CNR2-5-e1545-s001.pdf]

Title: Genetic features of precursor B-cell phenotype Burkitt leukemia with IGH-*MYC* rearrangement

Supplementary methods

Sample preparation and whole exome sequencing

Using the QIAamp DNA Mini Kit (Qiagen), the tumor and germline DNA were extracted from blast cells at diagnosis and peripheral blood during remission, respectively. Library construction was performed with SureSelect Human All Exon Kit v6 (Agilent Technology). Enriched fragment libraries were then sequenced on the HiSeq 2500.

Short read mapping and variant detection

Short reads obtained from the sequencer were processed, mapped, and analyzed by modifying a previous report.[1] In brief, the paired-end reads were first trimmed by removing library adapters and low-quality bases at the ends and then aligning to the hs37d5 sequence (GRCh37 and decoy sequences) using the Burrows-Wheeler Aligner MEM. Uniquely mapped read pairs were selected to make SAM and BAM files, followed by removal of PCR duplicates, local realignment, and recalibration of map quality scores. The mean coverage of diagnostic and remission samples was 108.9 and 109.6, respectively. Multi-sample calling with diagnostic and remission samples using the Genome Analysis Toolkit (GATK) HaplotypeCaller was employed for the detection of mutations. Annotations of altered sites were made using the ANNOVAR software based on GRCh37 with annotation databases.

Identification of germline variants of cancer-predisposing genes

A total of 607 genes, including 110 genes regarded as following a dominant inheritance pattern and 52 genes with recessive inheritance, were selected for analysis from the gene lists from previous reports.[2,3] First, to detect the rare variants, variants with > 0·1% population frequency in the 1000 Genomes Project database and the Human Genetic Variation Database (HGVD), a reference database of genetic variations in the Japanese population, were excluded.[4,5] Pathogenicity of detected variants was manually reviewed using the online databases including NCBI ClinVar, Leiden Open Variation Database, and Catalogue of Somatic Mutations in Cancer (COSMIC).

Droplet digital PCR

The ddPCR analysis was performed using QX-200 (Bio-Rad, Hercules, CA,

USA) to determine the precise frequency of *KRAS* mutation alleles, according to the manufacturer's protocol. A ready-made primer, ddPCR™ Mutation Assay: KRAS p.G13D c.38G>A (Bio-Rad, Hercules, CA, USA), was used. Each sample was triplicated and the average was calculated as the mutation allele frequency.

References

1. Fukawatase Y, Toyoda M, Okamura K, et al. Ataxia telangiectasia derived iPS cells show preserved x-ray sensitivity and decreased chromosomal instability. Scientific reports 2014;4:5421.
2. Grobner SN, Worst BC, Weischenfeldt J, et al. The landscape of genomic alterations across childhood cancers. Nature 2018;555(7696):321-327.
3. Zhang J, Walsh MF, Wu G, et al. Germline Mutations in Predisposition Genes in Pediatric Cancer. The New England journal of medicine 2015;373(24):2336-2346.
4. Higasa K, Miyake N, Yoshimura J, et al. Human genetic variation database, a reference database of genetic variations in the Japanese population. J Hum Genet 2016;61(6):547-553.
5. Narahara M, Higasa K, Nakamura S, et al. Large-scale East-Asian eQTL mapping reveals novel candidate genes for LD mapping and the genomic landscape of transcriptional effects of sequence variants. PLoS One 2014;9(6):e100924.

Supplementary Table S1. Flow cytometry findings at diagnosis

B-cell antigens	(%)	T-cell antigens	(%)
cyCD22	98.6	cyCD3	0.0
c-CD79a	99.7	CD7	0.8
CD19	99.0	CD2	0.6
CD10	97.9	CD3	0.3
CD24	99.1	CD4	0.3
cyμ	91.5	CD5	1.0
CD22	98.8	CD8	0.3
CD20	2.0	TCR α/β	0.7
CD21	0.8	CD1a	0.0
μ	12.4	TCR γ/δ	0.2
κ	0.2		
λ	2.8		
**Other antigens**	**(%)**	**Myelocytes, monocytes, and megakaryocytic antigens**	**(%)**
cyTDT	0.0	cy-MPO	0.5
HLA-DR	99.5	CD11b	1.4
CD58	0.9	CD13	0.8
CD34	0.0	CD14	0.3
CD99	1.0	CD15	0.2
7.1	0.2	CD33	0.5
CD56	0.1	CD64	0.2
CD244	0.4	CD65	0.2
CRLF2	0.1	CD117	0.0
CD38	99.8	CD36	0.3
CD133	0.0	CD41	0.0
CD27	0.2	CD42b	0.2
		CD61	0.2
		CD235a	4.4
		CD66c	0.1

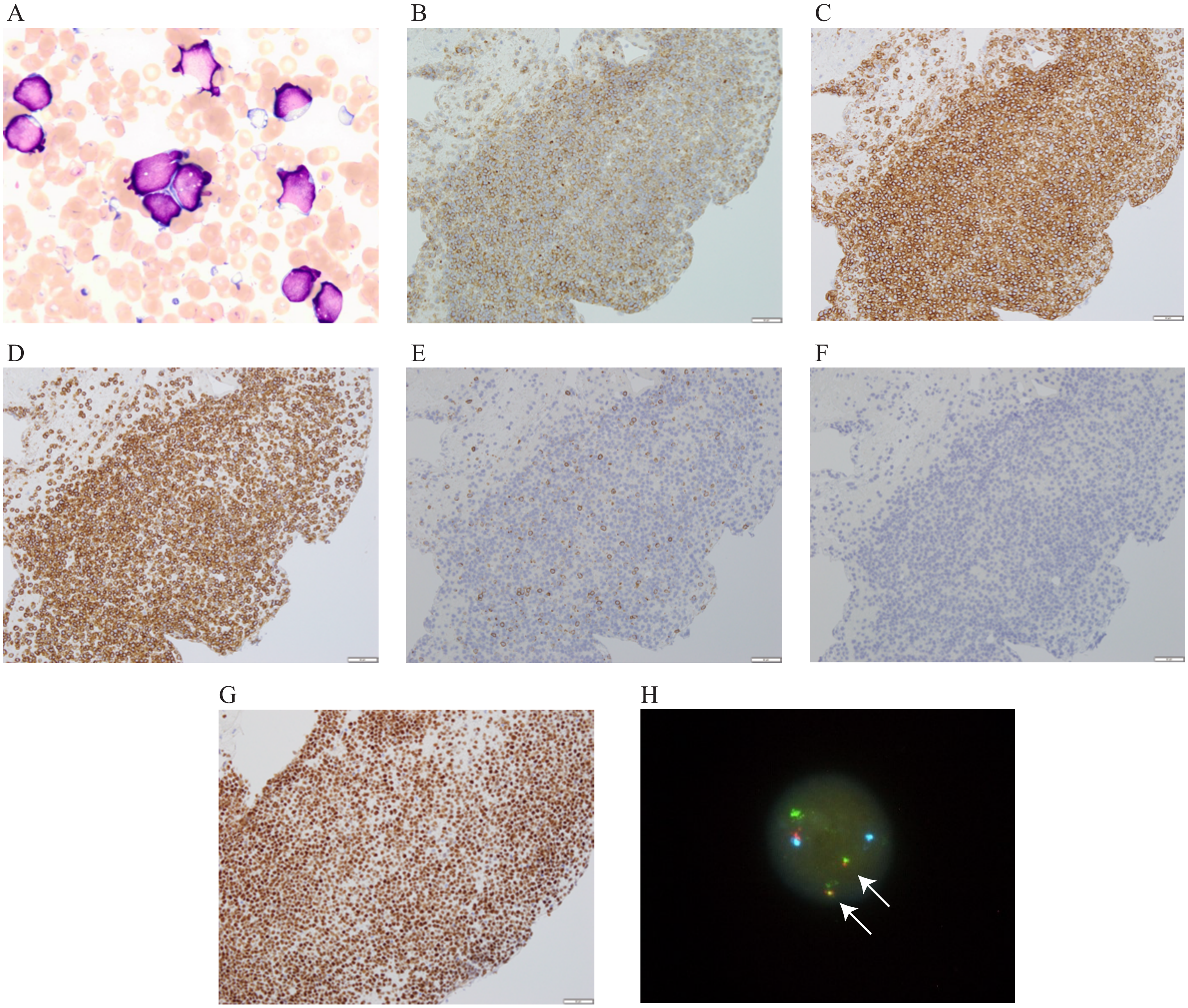

Supplementary Figure S1. Morphologic, immunocytologic, and cytogenetic features of leukemic blasts
Peripheral blood smear reveals increased blast cells with basophilic cytoplasm containing vacuoles (A). In immunostaining, the blasts were positive for CD10 (B), CD19 (C), CD79a (D), and c-Myc (G), and negative for CD20 (E) and TdT (F). Fusion FISH of IGH-*MYC* was potive (H).

FBXO11 NM_001190274:exon14

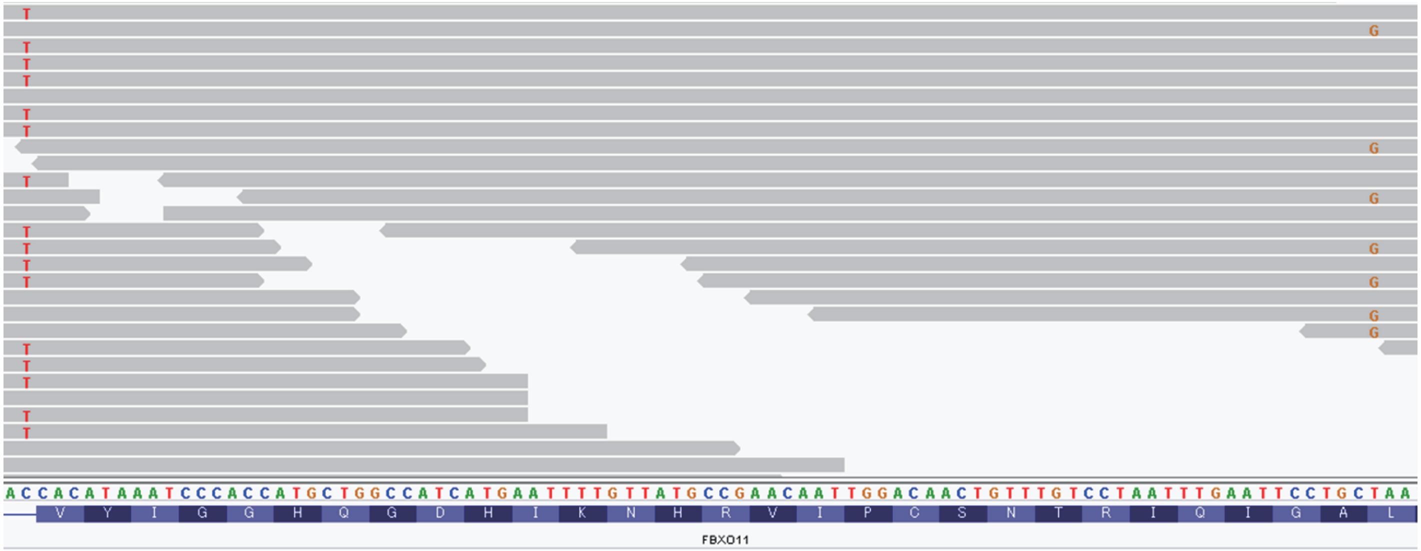

Supplementary Figure S2. Genomic location of the two FBXO11 mutations

Visual screening using the IGV viewer revealed that the two mutations in ***FBXO11*** were on different alleles (in trans).
